# The relationship between university teachers' professional identity and teaching innovation behavior: the mediating role of teacher professional capital and the moderating effect of team climate

**DOI:** 10.3389/fpsyg.2026.1863343

**Published:** 2026-07-17

**Authors:** Zenghui Zhou, Wenbin Luo

**Affiliations:** 1 College of Educational Science, Hunan Normal University, Changsha, Hunan, China; 2 College of Art, Hunan University of Information Technology, Changsha, Hunan, China

**Keywords:** professional identity, teacher professional capital, teaching innovation behavior, team climate, university teachers

## Abstract

**Background:**

Amid continuing change in higher education, innovative teaching by university teachers has become central to improving educational quality and advancing universities' development. Previous research has linked professional identity, teacher professional capital, and team climate to teaching innovation behavior, but less is known about the resource-based mechanism and contextual boundary conditions underlying these associations.

**Purpose:**

Based on professional identity theory and conservation of resources theory, this study intends to explore the association between university teachers' professional identity and teaching innovation behavior, and to further analyze the roles played by teacher professional capital and team climate in this context.

**Methods:**

A questionnaire survey was conducted, and 687 valid responses were collected. All statistical analyses were conducted using SPSS 27.0 and AMOS 27.0.

**Results:**

The structural equation modeling results indicated positive associations between professional identity and both teacher professional capital and teaching innovation behavior. Teacher professional capital served as a partial mediator in the relationship between professional identity and teaching innovation behavior. Further analyses showed that team climate significantly moderated the relationships among professional identity, teaching innovation behavior, and teacher professional capital.

**Conclusion:**

The findings highlight the importance of professional identity, professional resource accumulation, and supportive team climate in understanding university teachers' teaching innovation behavior.

## 1 Introduction

As digital technologies, especially artificial intelligence, become embedded in higher education, university teachers are under growing pressure to upgrade their pedagogical expertise and rethink how innovation is enacted in teaching

(Avidov-Ungar and Forkosh-Baruch, 2018; Zhang, 2023). At the same time, prevailing instructional models have transitioned from teacher-centered instruction to student-centered learning paradigms, and teachers are transitioning from “transmitters of knowledge” to “facilitators of learning” (Kim, 2025). This teaching model and the dual transformation of the teacher's role have placed higher demands on the teaching concepts, teaching methods, and teaching competencies of university teachers. This dual transformation also makes teaching innovation an important pathway through which university teachers adapt to changes in higher education.

Teaching innovation behavior refers to the novel and effective teaching practices, methods, or materials initiated or adopted by teachers in the classroom environment (Anderson et al., 2014). As a core manifestation of teachers' professional development and an important professional responsibility, teaching innovation behavior is not only an inevitable requirement for teachers to keep pace with the educational change and adapt to the modernization of education, but also a critical support for nurturing innovative talent in tertiary education (Anjali and Kaushal, 2017; Wang et al., 2025). Therefore, against the backdrop of ongoing change in higher education, examining university teachers' teaching innovation behavior and the factors associated with such behavior carries substantial theoretical significance and practical value.

Existing studies indicate that teaching innovation behavior does not emerge in isolation. Rather, it is associated with both individual and organizational factors. At the individual level, professional identity, teacher professional capital, teaching self-efficacy, intrinsic motivation, and emotional intelligence have all been found to be closely related to teaching innovation behavior (Klaeijsen et al., 2017; Li et al., 2025; Sachs, 2001; Tan et al., 2017; Wang, 2025). At the organizational level, participation climate, organizational learning, and collaborative culture have also been shown to be related to teaching innovation behavior (Naqshbandi et al., 2019; Putra et al., 2020; Yang et al., 2018). These empirical observations imply that teaching innovation behavior is not only associated with teachers' individual cognition and resources but also with the team context in which they work. However, these factors do not occupy the same theoretical position. Professional identity, teacher professional capital and team climate deserve special attention because they respectively correspond to the three key conditions for teaching innovation: role-value orientation, professional resource accumulation, and the immediate collaborative context.

Professional identity, which runs throughout teachers' career development, can provide stable intrinsic motivation and role guidance for instructional change, and has been positively associated with teaching innovation behavior (Sachs, 2001; Tan et al., 2017). Teacher professional capital is characterized as a collection of personal and collective resources, abilities, and practical wisdom possessed by teachers. Research has indicated a positive connection between teacher professional capital and teaching innovation behavior, and teacher professional capital provides an important resource basis for the implementation of innovative teaching (Hargreaves and Fullan, 2012; Liu and Zhang, 2024). As an important situational condition, team climate is related to teachers' psychological safety, peer support, and access to resource sharing, and has been positively associated with teaching innovation (Anderson and West, 1998). Therefore, placing these three factors within a unified analytical framework may provide a clearer understanding of the relationships surrounding teaching innovation behavior among university teachers.

Although prior studies have linked professional identity (Avidov-Ungar and Forkosh-Baruch, 2018), teacher professional capital (Liu and Zhang, 2024), and team climate (Bain et al., 2001) to teaching innovation behavior, several issues remain insufficiently addressed. First, the existing research mainly focuses on direct correlations, but pays less attention to how professional identity is related to teaching innovation behavior through resource-based mechanisms. Teacher professional capital may play this role as it reflects the knowledge, practical abilities, collaborative resources, and professional judgment accumulated by teachers. Second, teaching innovation is embedded in specific team contexts, yet prior research has not fully explained how team climate functions as a boundary condition in the associations among professional identity, teacher professional capital, and teaching innovation behavior. Third, the higher education system in China provides a meaningful environment for addressing these issues. Chinese universities are undergoing digital transformation, quality improvement reforms, and an increasing demand for the professional development of teachers. Against this backdrop, teaching innovation is often organized through departments, course groups, and teaching-research teams rather than carried out only as an individual activity. Therefore, examining professional identity, teacher professional capital, and team climate among university teachers in China may provide a more context-sensitive understanding of teaching innovation behavior.

Drawing on professional identity theory (Beijaard et al., 2004) and conservation of resources theory (Hobfoll, 1989), this study examines the connection between professional identity and teaching innovation behavior among university teachers in China, with teacher professional capital serving as a mediating resource mechanism and team climate as a contextual moderator. This design seeks to provide a more context-attuned view of the innovative teaching practices used by university teachers, and to offer practical implications for faculty development in higher education.

## 2 Review and hypothesis

### 2.1 Theoretical basis

The ideological roots of the professional identity theory can be traced back to Erikson's self-identity theory (Erikson, 1968), and the theory has been further developed and improved in the work of Ashforth and Mael (1989) and Holland et al. (1993). According to this theory, individuals gradually form an understanding and internalization of their own professional roles, professional values and norms of conduct through continuous interaction with the professional environment; this process provides important guidance for career choice, career commitment and career adaptation. For teachers, professional identity is not only reflected in the understanding and acceptance of the teacher role, but also in the positive affirmation of educational mission, professional responsibility and teaching value (Beijaard et al., 2004; Sachs, 2000). A robust sense of professional identity helps to enhance teachers' dedication, sense of responsibility and initiative in teaching, so that they display an increased tendency to explore alternative pedagogical methods, adopt new concepts, and actively engage in teaching innovation in practice. Therefore, the theory of professional identity provides an important perspective for understanding the link between university teachers' professional identity and teaching innovation behavior.

Conservation of resources theory, put forward by Hobfoll (1989), posits that individuals have an inherent motivation to acquire, preserve and build valuable resources, and the availability and loss of these resources are closely related to individuals' psychological states and behavioral choices. The theory argues that resources include not only material resources, but also personal characteristics, environmental conditions, energy resources and other forms of resources. When individuals have enough resources, they can better cope with stress, accept challenges and engage in proactive behaviors, and vice versa: they are more likely to fall into a defensive or avoidant state (Hobfoll, 2012). In educational and organizational behavior research, conservation of resources theory is often used to explain phenomena such as work input, career commitment, innovative behavior and coping with pressure (Crawford et al., 2010; Salmela-Aro et al., 2021). Within the context of higher education, teaching innovation often requires additional time, access to new knowledge, experimentation with new methods, and willingness to take risks; therefore, this is an effort that relies heavily on a variety of resource support. In this process, professional identity can be understood as a personal resource because it provides teachers with professional meaning, value commitment, and psychological resilience when facing the demands of teaching innovation. Teacher professional capital can be regarded as an important accumulation of professional knowledge, practical ability, collaborative relationship, educational wisdom and other resources. Team climate is an important external situation resource for teaching innovation behavior. Therefore, the conservation of resources theory offers a valuable lens for examining how professional identity, teacher professional capital, and team climate jointly operate to shape teaching innovation behavior. This theoretical framework posits that these three elements operate in concert to shape educators‘ innovative instructional practices.

Although self-determination theory, social cognitive theory, and job demands-resources model also offer useful perspectives on innovative teaching, the present study focuses on professional identity theory and conservation of resources theory because the central question concerns how teachers' professional identity is connected with teaching innovation through professional resources and team-based contextual conditions. This theoretical focus is therefore consistent with the proposed role-resource-context framework.

Drawing on prior research, our study integrates professional identity theory (Beijaard et al., 2004; Sachs, 2001) and conservation of resources theory (Hobfoll, 1989, 2012) to construct a role–resource–context framework for understanding university teachers' teaching innovation behavior. Professional identity theory explains why teachers' understanding of their professional role, educational mission, and professional value is associated with their orientation toward innovative teaching. Conservation of resources theory further explains how professional identity, as a personal resource, may be linked to teaching innovation behavior through the accumulation and mobilization of professional resources. In this framework, teacher professional capital is examined as a mediating resource mechanism linking professional identity and teaching innovation behavior, because it reflects teachers' accumulated professional knowledge, practical competence, collegial resources, and educational judgment. Team climate is positioned as a contextual boundary condition, because it represents the immediate collaborative environment in which teachers exchange ideas, receive feedback, share resources, and test new teaching approaches. In sum, this comprehensive framework delineates theoretically interrelated pathways, where professional identity may relate to teacher professional capital, teacher professional capital is associated with teaching innovation behavior, and team climate may moderate the strength of these connections.

### 2.2 Professional identity and teaching innovation behavior

Professional identity refers to how an individual views themselves in a professional environment, and it is formed through a commitment to one's professional abilities and responsibilities. Its development spans the entire career of an individual, involving the internalization of professional roles, values and behavioral norms (Tan et al., 2017). Individuals with stable professional identity often have a strong identity with the role and core values of their profession, and gain a sense of meaning and belonging from professional practice (Tan et al., 2017). This psychological state is further transformed into the inherent resilience and behavioral motivation to cope with career challenges. Accordingly, teacher professional identity has been viewed as an essential factor linked to professional development (Beijaard et al., 2004).

Earlier studies indicate that a robust sense of professional identity often acts as a driving force behind shifts and reforms in teaching practices, suggesting a meaningful relationship between university teachers' professional identity and their engagement in teaching innovation (Sachs, 2001). From the standpoint of professional identity theory, professional identity provides important intrinsic support for teaching innovation behavior. Through the lens of the conservation of resources theory, one may conceptualize professional identity as an individual-level resource capable of fostering teachers' involvement in teaching innovation behavior. A stronger sense of identity may be associated with teachers' understanding of the professional significance of teaching innovation, greater professional investment, internal motivation, teaching reflection, updated teaching concepts, and varied teaching methods (Beijaard et al., 2004). Existing research also supports this relationship. For example, Chun et al. (2025) found that language teachers who perceived themselves as learning facilitators rather than mere knowledge transmitters were more likely to integrate digital tools and innovative teaching methods into their classrooms. Their identity perceptions were directly associated with the frequency of teaching innovation behavior. Similarly, Teng and Yip (2025) found that university teachers who actively reconstructed their professional identity and regarded innovative practice as central to their role were more willing to experiment with AI-assisted instructional design and interactive classrooms.

### 2.3 Teacher professional capital as a mediating resource mechanism

Coined by Hargreaves and Fullan (2012), teacher professional capital denotes the aggregate professional assets that educators amass across their occupational pathways. These resources are mainly reflected in professional knowledge, practical skills, collaborative support and educational judgment, which are regarded as an important foundation for teachers to engage in professional practice and achieve sustainable development (Fullan, 2016; Reichenberg and Andreassen, 2018). As the core resource for teachers' professional development, teacher professional capital not only reflects the individual professional quality of teachers, but also reflects teachers' ability to mobilize and allocate resources in educational practice.

Earlier studies have illustrated a robust link between teacher professional identity and teacher professional capital (Hargreaves and Fullan, 2012). From the viewpoint of professional identity theory, a stronger sense of professional identity motivates teachers to invest more proactively in professional development (Douwe et al., 2000). From a conservation of resources theory perspective, teachers may use professional identity as an initial personal resource to invest in professional growth (Hobfoll, 1989). These investments help them accumulate teacher professional capital. On the one hand, relatively abundant teacher professional capital is associated with teachers' stronger sense of professional competence, professional value, and collegial belonging, which may be associated with a stronger identification with the teacher role. On the other hand, a stronger professional identity may be linked to greater proactiveness in professional growth, sustained learning, and the gathering of practical resources (Berger and Lê Van, 2018; OECD, 2016; Richter et al., 2021). This suggests that professional identity and teacher professional capital are interrelated throughout teachers' professional growth. In the study by Geijsel et al. (2009), teachers with stronger professional identity also tended to report higher levels of professional capital.

In addition, teacher professional capital has also been recognized as a crucial factor linked to innovative teaching behavior (Liu et al., 2020; Liu and Zhang, 2024). Guided by conservation of resources theory, the more resources an individual possesses, the better equipped they are to handle tasks that require high effort and involve high uncertainty (Hobfoll, 1989). Teaching innovation behavior is not a simple teaching adjustment; rather, it is a process that requires continuous investment in knowledge, skills, experience, and collaborative support. When implementing teaching innovation, teachers often need to update teaching concepts, try new teaching methods, and make professional judgments in complex teaching situations, which places high demands on teachers' professional resources. Teacher professional capital consists of the professional resources that teachers have accumulated in terms of professional knowledge, practical skills, collaborative support, and educational judgment; as such, it provides the necessary foundation for instructional innovation (Hargreaves and Fullan, 2012). For instance, Liu and Zhang (2024) reported a significant positive association between teacher professional capital and teaching innovation behavior: the richer the teacher professional capital, the more likely teachers were to exhibit a higher level of teaching innovation behavior.

Taken together, these arguments imply that teacher professional capital could function as a mediating resource pathway between professional identity and innovative teaching behavior. Grounded in educators' occupational roles and pedagogical values, professional identity may function as an personal resource that corresponds to enhanced teacher professional capital. Teacher professional capital, in turn, represents the professional resources that teachers accumulate and apply in their practice. In this sense, greater teacher professional capital may further be associated with more active teaching innovation behavior.

### 2.4 The moderating role of team climate

Team climate is conceptualized as the relatively stable psychological environment formed within a team that is related to members' behavior and attitudes, typically characterized by trust and support, peer assistance, resource sharing, and tolerance for innovation (Cohen et al., 2009). In the present study, “team” refers not to the university as a whole, but to the immediate work unit in which faculty members regularly collaborate on shared teaching, research, or administrative tasks. In the university setting, team climate reflects not only the level of support provided by the organizational environment in which faculty members work, but also the opportunities for collaboration, feedback resources, and emotional support available to them in their teaching practice. Previous research has demonstrated that a positive team climate is related to members' sense of safety, belonging, and willingness to collaborate, thereby providing favorable external conditions for professional growth and behavioral performance (Han et al., 2023; Melesse and Belay, 2022; Peng et al., 2025). Consequently, team climate may be conceptualized as a significant situational factor pertinent to the professional development and pedagogical engagement of university faculty (El-Adawy et al., 2022). Although Chinese universities are often characterized as loosely coupled systems at the organizational level, their proximal work units still involve recurring interaction, shared goals, and varying degrees of task interdependence (Anderson and West, 1998; Bender and Laverty, 2020; Weick, 1976; Yan and Guan, 2020). In the present study, “team” refers to the immediate work unit—such as an administrative department, a research lab, or a teaching module team. Within these units, participative safety does not presuppose the absence of hierarchy; members may still feel safe to voice ideas when professional trust and collegial norms are present, even in China's hierarchically organized university settings (Anderson and West, 1998). Therefore, the concept of team climate remains meaningful in higher education when applied to such immediate collaborative units rather than to the institution as a whole. From this standpoint, a facilitative team climate, encompassing the degree to which members perceive psychological safety in sharing insights and engaging in collaborative discourse, may determine whether educators can efficiently activate their professional assets toward teaching innovation behavior (Anderson and West, 1998).

Grounded in conservation of resources theory, individuals are more likely to transform their existing intrinsic motivation and professional resources into proactive behavior when they are situated in contexts with greater external support (Hobfoll, 1989). For university teachers, pedagogical innovation often involves a high degree of uncertainty and substantial investment; it requires not only a strong sense of professional identity and ample teacher professional capital, but also a supportive, responsive, and inclusive team environment (Bain et al., 2001; Eisenbeiss et al., 2008). Conversely, if team support is insufficient and collaboration is weak, even teachers who possess a strong professional identity or ample teacher professional capital may find their teaching innovation behavior constrained (Hargreaves and Fullan, 2012; Liu and Zhang, 2024). Thus, team climate may moderate the positive associations among professional identity, teacher professional capital, and teaching innovation behavior.

The association between professional identity and teacher professional capital may also be moderated by the team climate. Teachers with a robust professional identity tend to invest more heavily in professional development and engage proactively in teaching enhancement and professional learning (Beijaard et al., 2004; Tan et al., 2017). However, whether this investment can be further transformed into the accumulation of teacher professional capital depends largely on whether the team can provide a collaborative platform, shared resources, and supportive feedback. Existing research shows that the organizational atmosphere characterized by openness, trust, and mutual support is associated with more professional interaction and experience sharing among teachers, thus creating favorable conditions for the formation and accumulation of teacher professional capital (Hargreaves and Fullan, 2012; Knowles et al., 2015; Nahapiet and Ghoshal, 1998). Therefore, in the context of a more positive team climate, the positive association between professional identity and teacher professional capital tends to be stronger.

Additionally, team climate might serve as a moderating mechanism in the linkage between teacher professional capital and teaching innovation behavior. Teacher professional capital embodies the comprehensive resources teachers have accumulated in professional knowledge, practical competence, collaborative support, and educational judgment, and these resources provide an important foundation for teaching innovation behavior (Hargreaves and Fullan, 2012). However, whether resources can truly be transformed into innovative practice depends on the supportiveness of the team environment. In a positive team climate, teachers find it easier to test new ideas and refine their instructional designs through collaborative interaction. With the support of peers, they also perceive less risk in trying new methods, thereby enabling their existing teacher professional capital to be translated more effectively into innovative teaching practice (Cohen et al., 2009). By contrast, in an environment lacking team support, even teachers with relatively high levels of teacher professional capital may reduce innovative practice because of inadequate feedback, collaboration, and tolerance (Liu and Zhang, 2024). Therefore, team climate may strengthen the positive association between teacher professional capital and teaching innovation behavior.

### 2.5 Hypothesis

**Figure 1:**
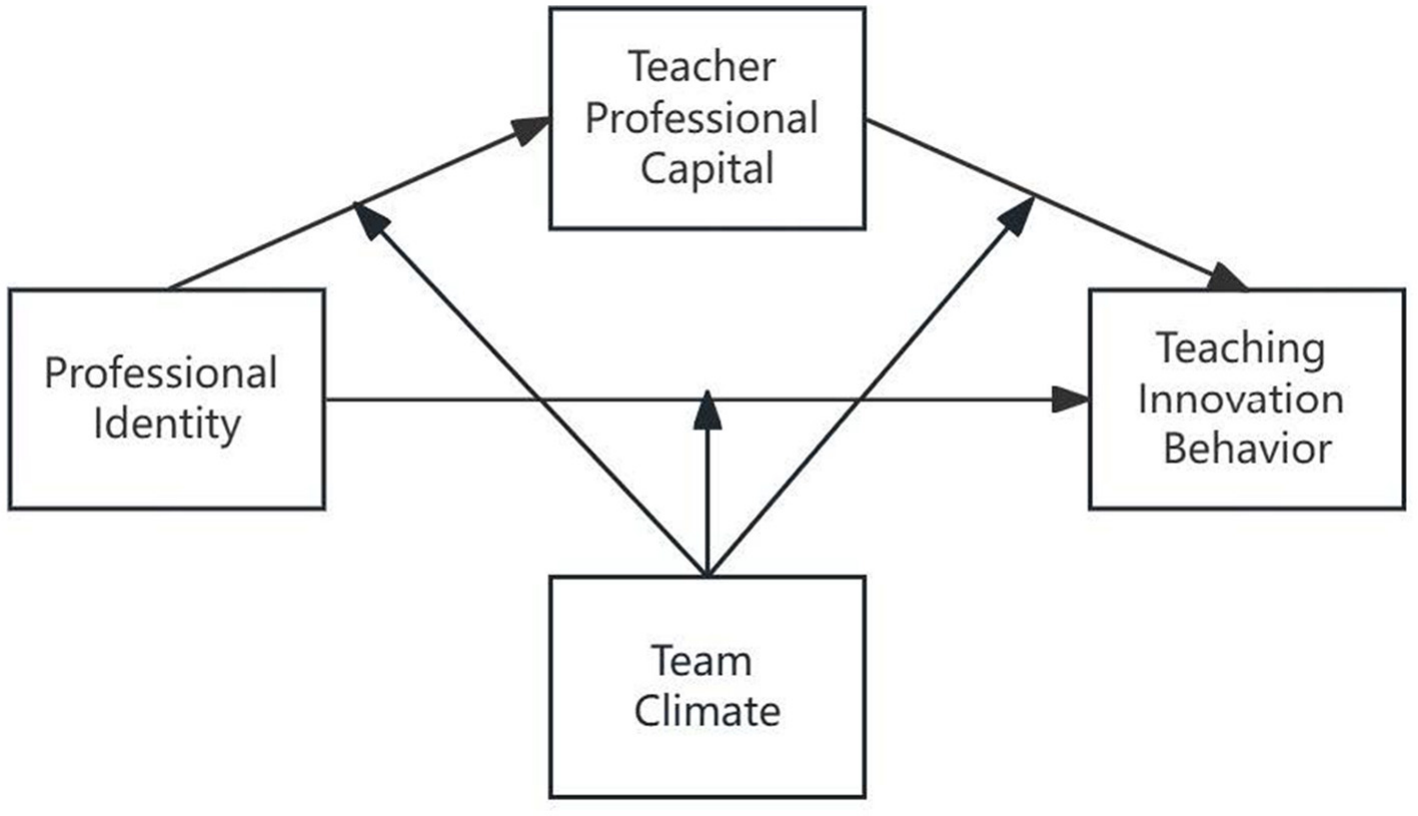
Proposed mediation and moderation model.

This study draws on professional identity theory and conservation of resources theory. Drawing on the theoretical discussions and empirical findings reviewed above, this study proposes that professional identity is associated with teaching innovation behavior both directly and indirectly through teacher professional capital. In addition, team climate is expected to function as a contextual boundary condition in these associations. Accordingly, this study constructs a theoretical model as shown in Figure 1 and proposes the following hypotheses:

H1: Teachers' professional identity is significantly and positively associated with teaching innovation behavior.
H2: Teachers' professional identity is significantly and positively associated with teacher professional capital.
H3: Teacher professional capital is positively associated with teaching innovation behavior.
H4: Teacher professional capital mediates the association between professional identity and teaching innovation behavior.
H5: Team climate significantly acts as a moderator of the relationship between teachers' professional identity and teaching innovation behavior.
H6: Team climate significantly acts as a moderator of the relationship between teachers' professional identity and teacher professional capital.
H7: Team climate significantly acts as a moderator of the relationship between teacher professional capital and teaching innovation behavior.

## 3 Research method

### 3.1 Sample and data collection

From March to April 2026, an online survey of university teachers was conducted via Wenjuanxing (https://www.wjx.cn/) at three universities in China. The three universities were located in Hunan, China, and represented different institutional types. The sampling frame consisted of full-time university teachers who were formally employed by the three participating universities during the data collection period and were involved in teaching or teaching-related work. Administrative staff, part-time teachers, visiting teachers, and teachers who were on long-term leave during the survey period were not included in the sampling frame. To protect institutional anonymity, they are referred to as University A, University B, and University C. University A was a public comprehensive university, University B was a public normal university, and University C was a public science and engineering university. Although the sample was drawn from one regional context, the inclusion of different institutional types helped improve sample diversity. The number of questionnaires distributed and valid responses obtained varied across universities, but all three institutions contributed valid responses to the final sample.

A multistage random sampling strategy was used to obtain the sample. After the research team contacted each university and obtained approval from the relevant administrative departments, lists of eligible teachers were obtained and participants were selected using random-number generation. With assistance from each institution, the study purpose, the voluntary nature of participation, and the principles of anonymity and confidentiality were explained to teachers in the survey setting. The questionnaire link was sent only to randomly selected teachers, and each questionnaire was assigned a unique identification code to strictly control respondent identity. The valid responses were then used for subsequent statistical analyses.

To limit possible common method bias, we adopted several design procedures before data collection. Participants completed the questionnaire voluntarily and anonymously, and we clearly stated that all answers reflected personal views rather than correct or incorrect choices. The questionnaire instructions emphasized that responses would be used only for academic research. Furthermore, we arranged the items measuring different constructs into separate sections, to reduce respondents' tendency to infer direct links among the variables.

Following Kline (2018) sample-size recommendation, at least 10 respondents were recommended for each questionnaire item. Because the survey instrument comprised 60 items, and an attrition rate of approximately 20% was anticipated, the minimum target sample size was set at 720 participants (60 × 10, plus 20% attrition). In total, 741 questionnaires were distributed and all were returned. After data screening, 54 questionnaires were removed because of missing data or extreme response patterns, such as selecting the same response option for all items. Consequently, 687 valid questionnaires were retained for the final analysis, yielding an effective response rate of 92.71%.

In terms of institutional distribution, 274 questionnaires were distributed at University A, 241 at University B, and 226 at University C. Because all distributed questionnaires were returned, unit non-response was limited in this study. The main concern was unusable-response bias caused by missing data or extreme response patterns. To address this issue, invalid questionnaires were excluded according to predefined screening criteria. However, because detailed information on teachers who did not provide usable responses was limited, potential non-response or unusable-response bias cannot be fully ruled out and should be considered when interpreting the findings.

Table 1 lists the demographic characteristics of 687 participants. There were 278 men (40.5%) and 409 women (59.5%). Most participants were aged 31–40 years (41.8%). The largest proportion had 4–6 years of teaching experience (24.6%). Most participants held a master's degree (58.7%), and the most common academic rank was lecturer (54.4%).

**Table 1:** Demographic characteristics of the sample.

Demographic characteristics	Category	Amount	Percentage (%)	*t/F*	*p*
Gender	Men	278	40.5	−4.576	< 0.001
	Women	409	59.5		
Age	≤ 30	130	18.9	5.122	0.002
	31–40	287	41.8		
	41–50	169	24.6		
	≥51	101	14.7		
Teaching experience	1–3	143	20.8	41.164	< 0.001
	4–6	169	24.6		
	7–10	133	19.4		
	11–15	155	22.6		
	≥16	87	12.7		
Educational qualifications	Doctor	192	27.9	3.316	0.011
	Master's degree	403	58.7		
	Undergraduate degree	92	13.4		
Professional title	Teaching Assistant	119	17.3	1.611	0.186
	Lecturer	374	54.4		
	Associate Professor	112	16.3		
	Professor	82	11.9		

The one-way analysis results presented in Table 1 indicate that gender, age, teaching experience, and educational background showed statistically significant differences in the study variables, whereas professional title did not. To reduce potential confounding from demographic variables, gender, age, teaching experience, and educational background were included as control variables in the subsequent SEM and moderation analyses. This method can more powerfully examine the relationship between professional identity, teachers' professional capital, team climate and teaching innovation behavior.

### 3.2 Measurement instruments

#### 3.2.1 Professional identity

This study employed the Professional Identity Five-Factor Scale (PIFFS) developed by Tan et al. (2017) to measure university teachers' professional identity. This instrument encompasses 25 items distributed across five facets: familiarity with occupational practices, exposure to the field, identification with an expert exemplar, domain-specific self-efficacy, and inclination toward a specific vocation. Within the facet of inclination toward a specific vocation, this instrument contains a single dichotomous item requiring an affirmative or negative response, while all other entries are evaluated using a five-point Likert format. Based on the measurement reliability and validity assessment, five items with relatively low standardized factor loadings (Q2, Q6, Q7, Q22, and Q24), including the dichotomous item Q24, were removed, and 20 items were retained. The sampling adequacy was acceptable (KMO = 0.970, Bartlett's test of sphericity *p* < 0.001). The overall Cronbach's α was 0.954, showing satisfactory psychometric properties.

#### 3.2.2 Teaching innovation behavior

University teachers' teaching innovation behavior was assessed using the Teaching Innovation Scale developed by Liu et al. (2016). The scale includes five items that capture teachers' tendency to try out new instructional ideas and to employ varied teaching methods or technologies to stimulate student engagement. All items were scored on a five-point Likert scale from 1 (strongly disagree) to 5 (strongly agree), and higher composite scores indicated stronger teaching innovation. Evidence from Chinese samples supports the reliability of this measure (Zhao and Huang, 2025). The sampling adequacy was acceptable (KMO = 0.884, Bartlett's test of sphericity *p* < 0.001). The overall Cronbach's α was 0.903, showing satisfactory psychometric properties.

#### 3.2.3 Teacher professional capital

This study employed Fullan (2016) Teacher Professional Capital Survey to assess university teachers' professional capital. The instrument includes three dimensions: human capital, social capital, and decisional capital, and contains 16 items. Responses were rated on a seven-point scale, ranging from 1 (strongly disagree) to 7 (strongly agree), with higher total scores indicating higher levels of teacher professional capital. The scale has been validated in Chinese samples and exhibits good internal consistency (Liu et al., 2020). The sampling adequacy was acceptable (KMO = 0.969, Bartlett's test of sphericity *p* < 0.001). The overall Cronbach's α was 0.958, showing satisfactory psychometric properties.

#### 3.2.4 Team climate

This study employed the Team Climate Scale developed by Anderson and West (1998) and adapted by Kivimäki and Elovainio (1999). In this research, “team” refers to the immediate work unit in which university teachers regularly collaborate on shared teaching, research, or administrative tasks. Accordingly, respondents were asked to evaluate the climate of the team or work unit with which they most closely interacted in their daily work. The scale contains 14 items. Each item was rated on a five-point Likert scale from 1 (strongly disagree) to 5 (strongly agree), with higher scores indicating a more favorable team climate. The scale has been validated in Chinese samples and demonstrates good internal consistency (Huang et al., 2024). The sampling adequacy was acceptable (KMO = 0.970, Bartlett's test of sphericity *p* < 0.001). The overall Cronbach's α was 0.956, showing satisfactory psychometric properties.

### 3.3 Statistical analysis

All analyses were conducted using SPSS 27.0 and AMOS 27.0. Descriptive statistics, normality tests, and Pearson correlations were first performed. Reliability was assessed using Cronbach's α and composite reliability, while convergent and discriminant validity were examined using AVE, the Fornell–Larcker criterion, and HTMT ratios. Common method bias was examined using Harman's one-factor test and the unmeasured latent method construct approach. Structural equation modeling (SEM) was then used to test the mediation model, and bootstrap analysis with 5,000 resamples was conducted to examine the indirect effect. Moderation effects were tested using the SPSS PROCESS macro, with relevant demographic variables included as covariates.

## 4 Results

### 4.1 Measurement model, reliability, and validity testing

As shown in Table 2, the evaluated model exhibited satisfactory psychometric performance. Following Fornell and Larcker (1981), the quality of the measurement model was assessed based on standardized factor loadings, composite reliability (CR), and average variance extracted (AVE). The standardized factor loadings of the retained items ranged from 0.547 to 0.834. Although 0.708 is commonly regarded as an ideal benchmark for item loadings, indicators with loadings between 0.40 and 0.70 may be retained when the corresponding constructs show acceptable CR and AVE values. In the present study, no retained item had a loading below 0.40. Internal consistency was further supported by the reliability results, with Cronbach's α values ranging from 0.903 to 0.958 and CR values ranging from 0.904 to 0.958, all exceeding the recommended threshold of 0.70 (Leguina, 2015). Convergent validity was also established, as the AVE values ranged from 0.509 to 0.653, all above the recommended cutoff of 0.50 (Fornell and Larcker, 1981; Leguina, 2015). Discriminant validity was examined using both the Fornell-Larcker criterion and the heterotrait-monotrait ratio (HTMT). As shown in Table 3, the square root of the AVE for each construct exceeded its correlations with the other constructs, indicating satisfactory discriminant validity according to the Fornell-Larcker criterion (Fornell and Larcker, 1981). In addition, the HTMT values presented in Table 4 ranged from 0.659 to 0.801, all below the recommended threshold of 0.85, which further supports discriminant validity among the constructs (Henseler et al., 2014). Collectively, the findings reveal that the assessed construct exhibited adequate internal consistency, convergent and discriminant validity.

**Table 2:** Reliability and validity of the model.

Constructs	Items	Loadings	Cronbach's α	CR	AVE
PI	PI1	0.615	0.954	0.953	0.509
	PI3	0.690			
	PI4	0.732			
	PI5	0.744			
	PI8	0.753			
	PI9	0.670			
	PI10	0.763			
	PI11	0.778			
	PI12	0.789			
	PI13	0.790			
	PI14	0.804			
	PI15	0.733			
	PI16	0.719			
	PI17	0.753			
	PI18	0.735			
	PI19	0.713			
	PI20	0.685			
	PI21	0.547			
	PI23	0.588			
	PI25	0.558			
TIB	TIB1	0.821	0.903	0.904	0.653
	TIB2	0.819			
	TIB3	0.829			
	TIB4	0.812			
	TIB5	0.758			
TPC	TPC1	0.648	0.958	0.958	0.586
	TPC2	0.723			
	TPC3	0.733			
	TPC4	0.748			
	TPC5	0.767			
	TPC6	0.799			
	TPC7	0.780			
	TPC8	0.815			
	TPC9	0.597			
	TPC10	0.759			
	TPC11	0.820			
	TPC12	0.783			
	TPC13	0.813			
	TPC14	0.811			
	TPC15	0.801			
	TPC16	0.792			
TC	TC1	0.696	0.956	0.957	0.608
	TC2	0.792			
	TC3	0.755			
	TC4	0.787			
	TC5	0.800			
	TC6	0.805			
	TC7	0.815			
	TC8	0.834			
	TC9	0.637			
	TC10	0.816			
	TC11	0.805			
	TC12	0.779			
	TC13	0.778			
	TC14	0.768			

PI, professional identity; TIB, teaching innovation behavior; TPC, teacher professional capital; TC, team climate.

**Table 3:** Discriminant validity based on the Fornell-Larcker criterion.

Construct	PI	TIB	TPC	TC
PI	**0.713**			
TIB	0.751	**0.808**		
TPC	0.681	0.669	**0.766**	
TC	0.636	0.615	0.698	**0.780**

The bold diagonal values represent the square roots of AVE. The off-diagonal values represent correlations among constructs. PI, professional identity; TIB, teaching innovation behavior; TPC, teacher professional capital; TC, team climate.

**Table 4:** Discriminant validity based on HTMT ratios.

Construct pair	HTMT
PI—TIB	0.801
PI—TPC	0.718
PI—TC	0.669
TIB—TPC	0.720
TIB—TC	0.659
TPC—TC	0.743

PI, professional identity; TIB, teaching innovation behavior; TPC, teacher professional capital; TC, team climate.

### 4.2 Descriptive statistics and correlation test

Table 5 reports the descriptive statistics and correlation analyses for the primary variables in this study. In order to comprehensively characterize the concentrated trend and distribution characteristics of each potential variable, the mean (M), standard deviation (SD), skewness (SK), and kurtosis (Kur) are reported herein. The results indicate that the skewness and kurtosis values of all variables were within acceptable ranges, suggesting that the data generally satisfied the assumption of normality. Correlation analysis showed that professional identity was significantly and positively correlated with teaching innovation behavior (*r* = 0.751, *p* < 0.001), teacher professional capital (*r* = 0.681, *p* < 0.001), and team climate (*r* = 0.636, *p* < 0.001). Teaching innovation behavior was also significantly and positively correlated with teacher professional capital (*r* = 0.669, *p* < 0.001) and team climate (*r* = 0.615, *p* < 0.001). In addition, teacher professional capital was significantly and positively correlated with team climate (*r* = 0.698, *p* < 0.001).

**Table 5:** The description and correlation of variables.

Constructs	M±SD	SK	Kur	PI	TIB	TPC	TC
PI	3.530 ± 0.788	−0.090	−0.446	1			
TIB	3.668 ± 0.916	−0.355	−0.460	0.751^***^	1		
TPC	4.748 ± 1.229	0.188	−0.677	0.681^***^	0.669^***^	1	
TC	3.575 ± 0.873	−0.157	−0.521	0.636^***^	0.615^***^	0.698^***^	1

^***^p < 0.001; PI, professional identity; TIB, teaching innovation behavior; TPC, teacher professional capital; TC, team climate.

### 4.3 Common method bias

Harman's one-factor test was used to evaluate common method bias. The first factor accounted for 46.423% of the total variance, which was below the 50% threshold recommended by Podsakoff et al. (2003). This result suggested that no single factor explained the majority of the variance. However, because Harman's one-factor test is only a preliminary diagnostic approach, this result alone cannot fully rule out the possibility of common method variance.

Subsequently, CFA was conducted to further examine the structural validity of the measurement model. As shown in Table 6, the overall model fit was acceptable: χ^2^ = 3582.990, df = 1,398, χ^2^/ df = 2.563, RMSEA = 0.048, SRMR = 0.043, GFI = 0.834, AGFI = 0.817, CFI = 0.927, IFI = 0.927, TLI = 0.923. All of these indicators met the standard requirements for structural equation modeling, supporting the conclusion that the model showed good overall fit and acceptable convergent validity. Furthermore, an unmeasured latent method construct (ULMC) model was tested through the inclusion of a common method factor. The results confirmed only marginal changes in fit indices relative to the baseline model, further confirming that common method bias was not a major concern in the present study (Marsh and Hocevar, 1985).

**Table 6:** CFA fit indices and comparison between Baseline and ULMC models.

Model	*χ^2^*(df)	*χ^2^*/ df	RMSEA	SRMR	GFI	AGFI	CFI	IFI	TLI
Reference value		< 3	< 0.080	< 0.080	>0.800	>0.800	>0.800	>0.800	>0.800
Baseline model	3582.990 (1398)	2.563	0.048	0.043	0.834	0.817	0.927	0.927	0.923
Method factor model	3449.947 (1398)	2.396	0.046	0.064	0.841	0.825	0.932	0.932	0.927
Model Fit Difference			ΔRMSEA	ΔSRMR	ΔGFI	ΔAGFI	ΔCFI	ΔIFI	ΔTLI
			0.002	0.021	0.007	0.008	0.005	0.005	0.004
Evaluation criterion			< 0.05	< 0.05	< 0.01	< 0.01	< 0.01	< 0.01	< 0.01

CMIN, chi-square value; DF, degrees of freedom; RMSEA, root mean square error of approximation; SRMR, standardized root mean square residual; GFI, goodness-of-fit index; AGFI, adjusted goodness-of-fit index; CFI, comparative fit index; IFI, incremental fit index; TLI, tucker-lewis index.

### 4.4 Collinearity test

To ensure the stability and interpretability of the model, collinearity among the independent variables was carefully examined. Tolerance values and variance inflation factors (VIFs) were computed in SPSS to assess the potential presence of multicollinearity. As reported in Table 7, all tolerance values were greater than 0.1, whereas the VIFs ranged from 2.055 to 2.391. In general, tolerance values above 0.1 suggest that serious collinearity is unlikely, and VIF values below 3.3 are typically regarded as acceptable (Hair et al., 2019). These results indicate that multicollinearity did not pose a substantial concern in the present study.

**Table 7:** Collinearity test of the structural model.

Constructs	Tolerance	VIF
PI	0.487	2.055
TPC	0.418	2.391
TC	0.465	2.152

PI, professional identity; TPC, teacher professional capital; TC, team climate.

### 4.5 Structural equation model results

**Figure 2:**
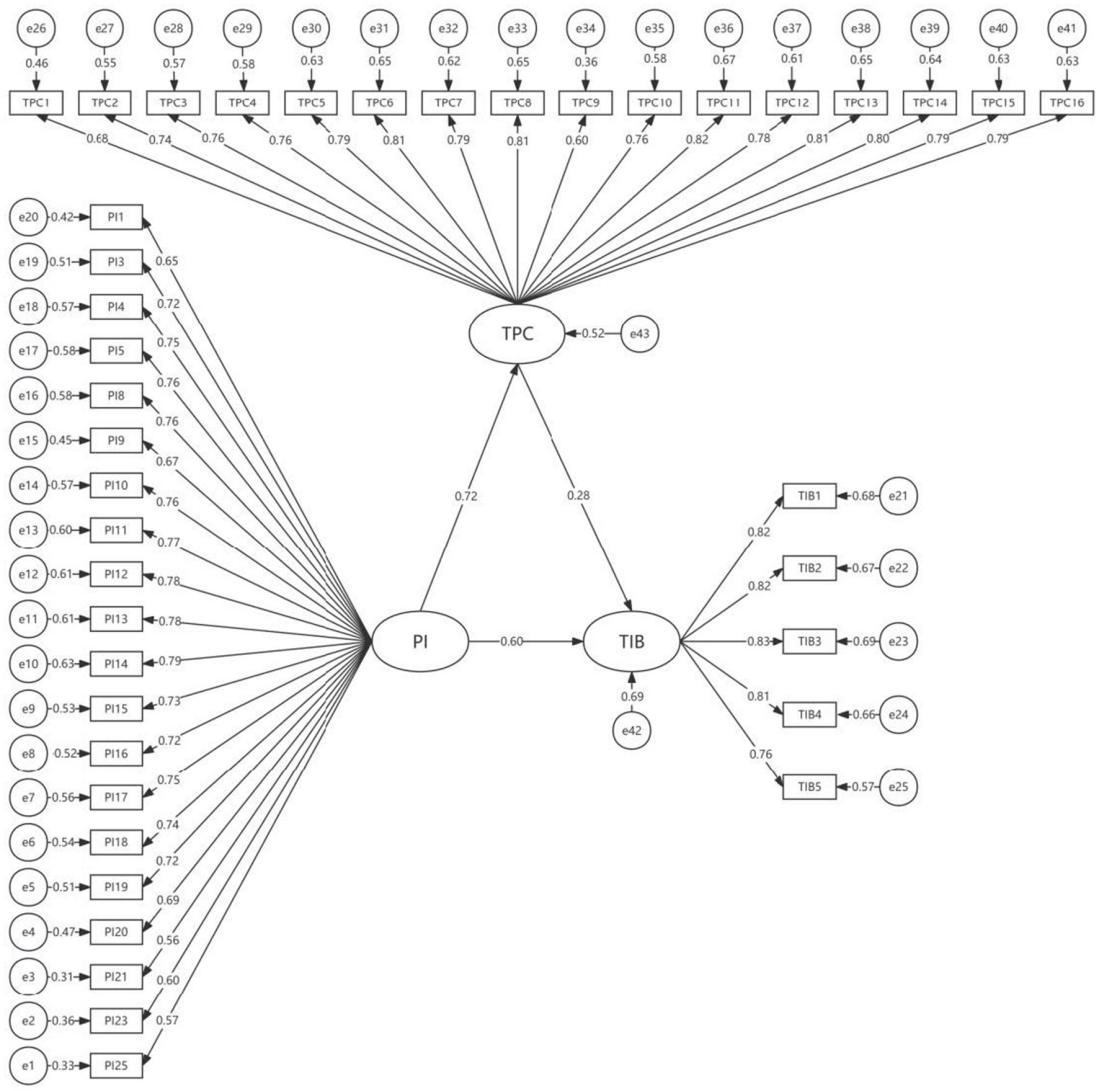
The structural model.

Based on acceptable results for the measurement model, factor loadings, reliability and validity, and CFA, we used AMOS to estimate the final structural model and examine the proposed hypotheses. Figure 2 presents the final SEM. The model showed a satisfactory match to the data, according to the goodness of fit indexes.

As shown in Table 8, professional identity was significantly and positively associated with teacher professional capital (β = 0.723, *p* < 0.001). Teacher professional capital was also significantly and positively associated with teaching innovation behavior (β = 0.285, *p* < 0.001). After teacher professional capital was included in the model, the direct association between professional identity and teaching innovation behavior remained significant (β = 0.599, *p* < 0.001). Therefore, H1, H2, and H3 were supported.

**Table 8:** Results of the mediation analysis.

Relationships	Effect	β	S.E.	C.R.	*P*-Value	Bias-corrected 95%CI
PI → TPC		0.723	0.099	13.019	< 0.001	[0.685,0.773]
TPC → TIB		0.285	0.033	6.901	< 0.001	[0.179,0.381]
PI → TPC → TIB	Indirect	0.206	0.035		0.009	[0.137,0.284]
	Direct	0.599	0.048		0.010	[0.495,0.704]
	Total	0.805				[0.763,0.851]

PI, professional identity; TIB, teaching innovation behavior; TPC, teacher professional capital; TC, team climate.

To assess the mediating role of teacher professional capital in the relationship between professional identity and teaching innovation behavior, we used the bootstrap procedure proposed by Preacher and Hayes (2008). The indirect effect was considered significant when the 95% bias-corrected confidence interval did not include zero. As shown in Table 8, teacher professional capital significantly mediated the association between professional identity and teaching innovation behavior, with an indirect effect of 0.206 and a 95% bias-corrected CI of [0.137, 0.284]. The total effect was also significant [β = 0.805, 95% CI = (0.763, 0.851)]. These findings suggest that teacher professional capital served as a partial mediator in the association between professional identity and teaching innovation behavior. Accordingly, H4 was supported.

### 4.6 Analysis of moderating effects

Table 9 presents the outcomes of the moderation analyses. With gender, age, teaching experience, and other demographic characteristics included as control variables, the professional identity × team climate interaction was significantly linked to higher teaching innovation behavior (β = 0.119, *p* < 0.001) and greater teacher professional capital (β = 0.462, *p* < 0.001). The interaction term between teacher professional capital and team climate was also significantly and positively associated with teaching innovation behavior (β = 0.152, *p* < 0.001). These findings indicate that team climate significantly moderated the relationships between professional identity and teaching innovation behavior, between professional identity and teacher professional capital, and between teacher professional capital and teaching innovation behavior. H5, H6 and H7 were all supported.

**Table 9:** The moderation effect.

Outcome variable	Predictor variable	β	SE	*t*	95% CI	
					LLCI	ULCI
TIB	PI × TC	0.119	0.033	3.589^***^	0.054	0.185
TPC	PI × TC	0.462	0.042	10.932^***^	0.379	0.545
TIB	TPC × TC	0.152	0.023	6.530^***^	0.106	0.197

^***^p < 0.001; PI, professional identity; TIB, teaching innovation behavior; TPC, teacher professional capital; TC, team climate.

**Figure 3:**
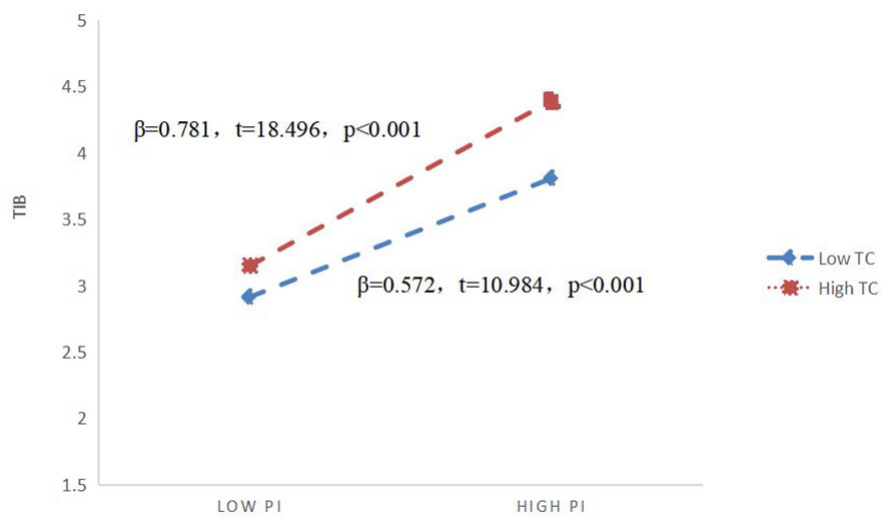
Moderating effect of TC on the association between PI and TIB.

**Figure 4:**
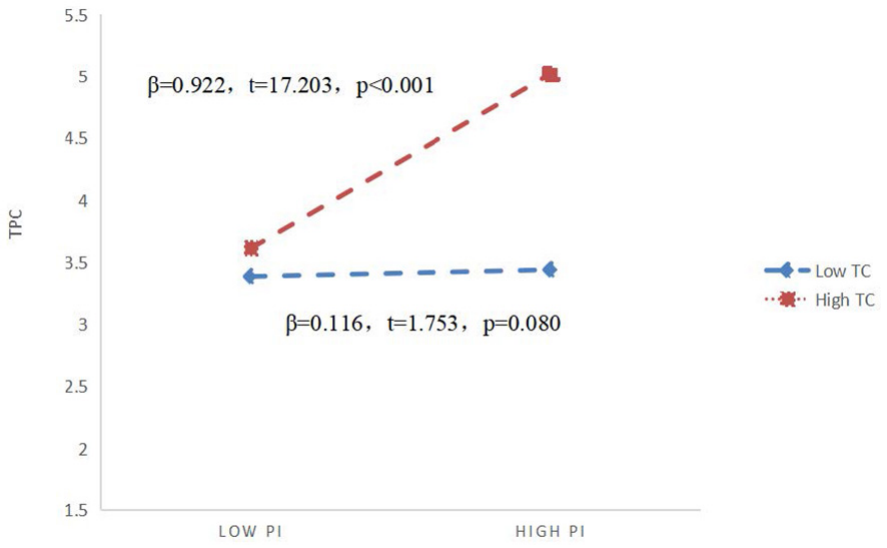
Moderating effect of TC on the association between PI and TPC.

**Figure 5:**
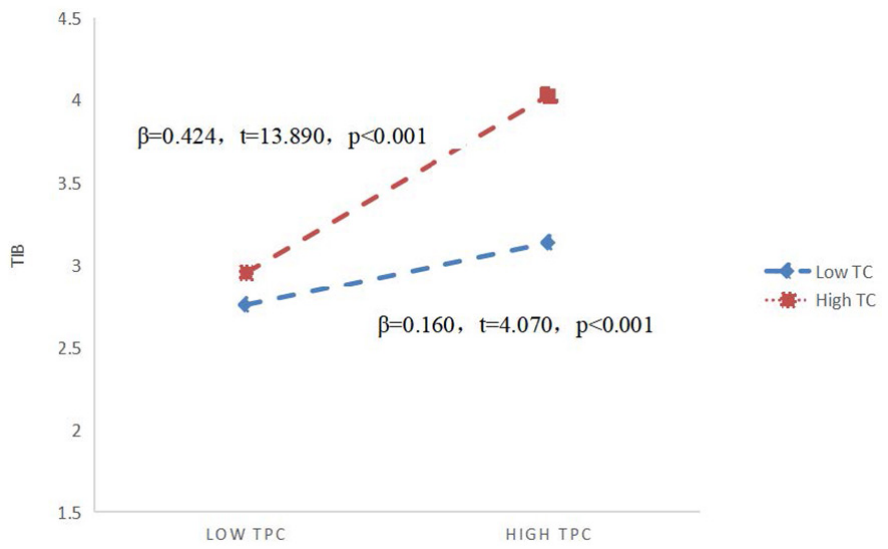
Moderating effect of TC on the association between TPC and TIB.

Simple slope plots (see Figures 3–5) showed that, in Figure 3, team climate significantly and positively moderated the association between professional identity and teaching innovation behavior. At high levels of team climate, the positive association between professional identity and teaching innovation behavior was stronger (β = 0.781, *t* = 18.496, *p* < 0.001), as indicated by the steeper slope. Under low team climate, although the positive association between professional identity and teaching innovation behavior remained statistically significant (β = 0.572, *t* = 10.984, *p* < 0.001), the effect size was much smaller and the slope was flatter than in the high-team-climate group. In other words, under a more positive team climate, professional identity showed a stronger positive association with teaching innovation behavior.

Figure 4 showed that team climate significantly and positively moderated the association between professional identity and teacher professional capital. Under a high team climate, professional identity was strongly and positively related to teacher professional capital (β = 0.922, *t* = 17.203, *p* < 0.001), with the steeper slope suggesting a stronger association. Under conditions of low team climate, however, the positive association between professional identity and teacher professional capital was not significant (β = 0.116, *t* = 1.753, *p* = 0.080). This suggests that when team climate is weak, the association between professional identity and teacher professional capital is weaker. Put differently, professional identity was more strongly associated with teacher professional capital under a positive team context.

Figure 5 further showed that team climate significantly and positively moderated the association between teacher professional capital and teaching innovation behavior. At high levels of team climate, teacher professional capital showed a stronger positive association with teaching innovation behavior (β = 0.424, *t* = 13.890, *p* < 0.001), as indicated by the steep slope. Under low team climate, although the positive association between teacher professional capital and teaching innovation behavior remained statistically significant (β = 0.160, *t* = 4.070, *p* < 0.001), the effect size was much smaller and the slope was flatter than in the high-team-climate group. In other words, teacher professional capital showed a stronger positive association with teaching innovation behavior under a positive team context.

## 5 Discussion

This study examined how professional identity, teacher professional capital, and team climate are connected with teaching innovation behavior among university teachers in China. Overall, these findings support the role-resource-context explanation. The professional identity theory explains the role-value basis of innovative teaching, indicating that teachers' identification with their professional role and educational mission is correlated with their orientation toward teaching innovation. Conservation of resources theory further explains how professional identity, as a personal resource, is linked to teaching innovation behavior through the accumulation and mobilization of professional resources. Within this framework, teacher professional capital represents accumulated professional resources, whereas team climate represents a contextual resource that may strengthen or weaken the conversion of these resources into teaching innovation behavior. Thus, the two theories jointly explain teaching innovation behavior as a process connected with professional identity, professional resources, and team-based collaboration.

The results showed a positive association between professional identity and teaching innovation behavior among university teachers. This finding is consistent with earlier studies showing that teachers with stronger professional identity tend to report more engagement in instructional change and innovative teaching practices (Liu and Zhang, 2024; Sachs, 2001; Tan et al., 2017). From the perspective of professional identity theory, teachers who have a clearer understanding of their professional roles, educational responsibilities, and teaching values may be more likely to view teaching innovation as part of their professional practice. This association is especially meaningful in the Chinese higher education context. Chinese universities are undergoing digital transformation and quality-oriented teaching reforms, and teaching innovation is often organized through departments, course groups, and teaching-research teams. In such a context, professional identity may not only reflect teachers' professional self-understanding, but also be closely connected with teachers' responses to institutional expectations and collective teaching reform. Therefore, this study extends prior research by placing professional identity within a broader role-resource-context framework rather than treating it as an isolated individual factor.

The results indicated significant positive correlations between professional identity and teacher professional capital, as well as between teacher professional capital and teaching innovation behavior. The SEM analysis further showed that teacher professional capital served as a partial mediator in the relationship between professional identity and teaching innovation behavior. The present result indicates that the association of professional identity with teaching innovation behavior may be interpreted as a role-resource mechanism. The theoretical framework of professional identity illuminates the role-value foundation underlying this mechanism, whereby educators with an enhanced perception of their occupational function and pedagogical mission are inclined to view teaching innovation behavior as constituting an element of their vocational responsibility. From a conservation of resources theory perspective, professional identity can also be understood as a personal resource. It may help teachers cope with the uncertainty and demands involved in instructional change and encourage them to invest in professional learning, collegial communication, reflective practice, and pedagogical improvement. Through these investments, teachers may accumulate the professional knowledge, practical competence, collaborative resources, and educational judgment that constitute teacher professional capital, which in turn supports teaching innovation behavior. This mechanism is especially relevant in Chinese universities, where teaching innovation is often embedded in departments, course groups, and teaching-research teams. In such a context, teacher professional capital represents a key resource pathway through which professional identity is associated with innovative teaching practice. Therefore, this study extends prior research by clarifying how professional identity and professional resources are connected in the teaching innovation process.

This study also found that team climate significantly moderated the associations linking professional identity, teacher professional capital, and teaching innovation behavior, and that these strengthening effects were more pronounced in the high team-climate group. This result accords with prior evidence from Bain et al. (2001) and Liu and Zhang (2024), both of which confirm that a positive team environment strengthens the link between individual resources and innovative behavior, thereby creating favorable conditions for resource conversion. This finding can be understood within a role-resource-context framework. Professional identity theory explains how teachers' understanding of their professional role and educational mission provides the value basis for innovative teaching. Conservation of resources theory further explains why this value basis is more likely to be translated into professional resource accumulation and innovative practice when teachers are situated in a supportive proximal collaborative unit. When the immediate work unit provides trust, feedback, shared resources, and tolerance for experimentation, teachers may have more opportunities to transform professional identity into professional learning, collegial exchange, and reflective practice, which are closely related to teacher professional capital and teaching innovation behavior. This mechanism is particularly relevant in Chinese universities, where teaching innovation is often embedded in departments, course groups, teaching-research teams, and other direct work units. In such settings, teachers' innovative practices are not only individual actions but are also shaped by collective expectations, institutional reform tasks, and collaborative routines. Therefore, team climate should not be viewed as a general background factor. It represents a proximal contextual condition under which professional identity and teacher professional capital are more strongly associated with teaching innovation behavior.

Overall, these findings have clarified the connections between professional identity, teacher professional capital, team climate, and teaching innovation behavior. Professional identity represents a personal resource grounded in teachers' role-value orientation, whereas teacher professional capital represents the professional resources through which this personal resource is translated into innovative teaching practice. Team climate represents the collaborative work environment in which these resources are exchanged and used. This integrated interpretation is especially meaningful in the Chinese higher education context. In many Chinese universities, teaching innovation is closely related to institutionalized teaching reforms, digital transformation, and collective teaching research activities. University teachers often develop and refine innovative teaching practices within departments, course groups, and teaching teams. Therefore, teaching innovation should not merely be regarded as an individual psychological outcome. A better way to understand it is to view it as a work-related process, in which professional identity, professional resources, and teamwork are closely interrelated.

## 6 Implication

### 6.1 Theoretical implication

The theoretical value of this study can be summarized in three main aspects. First, it develops a role-resource-context framework for understanding university teachers' teaching innovation behavior. This framework connects professional identity theory with conservation of resources theory by explaining how professional identity may be associated with teaching innovation through professional resource accumulation. To be more specific, one may construe professional identity as an individual-level resource capable of promoting educators‘ participation in teaching innovation behavior.

Second, this study clarifies the role of teacher professional capital. Rather than viewing teacher professional capital merely as a broad professional resource, this study considers it a mediating resource-related mechanism linking professional identity with teaching innovation behavior. This helps explain why professional identity is associated with teaching innovation from a resource-based perspective. This positioning responds to the need to explain not only whether professional identity is associated with teaching innovation behavior, but also through which professional resources this relationship may operate.

Third, this study highlights the contextual role of team climate. Teaching innovation in higher education is often embedded in departments, course groups, and teaching-research teams, especially in the Chinese university context. By examining team climate as a moderator, this study shows that the associations among professional identity, teacher professional capital, and teaching innovation behavior may differ across collaborative environments.

### 6.2 Practical implication

The findings provide several practical implications for supporting university teachers' teaching innovation behavior. At the individual level, teachers may benefit from strengthening their understanding of professional roles, educational values, and instructional responsibilities. Professional identity can be supported through reflective teaching practice, participation in teaching-research activities, and regular peer exchange. These activities may help teachers connect teaching innovation with their professional mission rather than viewing it only as an external requirement. In addition, teachers can accumulate teacher professional capital by updating disciplinary knowledge, improving pedagogical skills, learning digital teaching methods, and seeking feedback from colleagues and students.

At the school and departmental levels, universities should provide more structured support for teaching innovation. Departments can establish teaching innovation teams, peer mentoring groups, and regular teaching-research meetings. These platforms allow teachers to share course design experiences, discuss difficulties in classroom reform, and receive practical feedback from colleagues. Universities can also provide small-scale teaching innovation grants, workload recognition, and opportunities for interdisciplinary collaboration. Such measures may help teachers gain access to professional knowledge, collaborative resources, and practical support in their daily teaching work.

At the institutional level, teacher development should not treat teaching innovation solely as an individual responsibility. Universities can integrate professional identity, teacher professional capital, and team climate into a coherent faculty development system. For example, institutions may design training programs that combine value reflection, teaching skill development, peer collaboration, and digital pedagogy. They may also build evaluation and incentive mechanisms that recognize teachers' efforts in innovative teaching, tolerate reasonable trial and error, and encourage shared reflection within teaching teams. In this way, universities can create a more supportive work environment in which professional identity, professional resources, and collaborative support are integrated in practice.

### 6.3 Limitations and future directions

This study applied SEM and bootstrap procedures to analyze the proposed mediation model. However, the results should be considered in light of several limitations.

First, this survey adopted a cross-sectional survey design; therefore, the data reflect only the correlations among variables measured at a single time point, and strict causal inferences or conclusions about long-term trends cannot be drawn. Future research may adopt a longitudinal design. This would allow researchers to examine how professional identity, teacher professional capital, team climate, and teaching innovation behavior change over time and how their temporal associations unfold. Such designs may provide stronger evidence for understanding the temporal ordering among these variables.

Secondly, all data in this study were based on the teacher's self-reports. Although the common method bias test yielded acceptable results, a certain degree of subjective reporting bias may still exist. Future research can incorporate multi-source data, such as classroom observation, peer evaluation and student evaluations of teaching, to improve measurement accuracy and ecological effectiveness.

Third, although the SEM results showed acceptable model fit based on several widely used indices, including CFI, TLI, RMSEA, and SRMR, the GFI and AGFI values were lower than some conservative recommended thresholds. One possible explanation is that the model included multiple paths and a relatively high number of measured indicators, which may have contributed to this result. For this reason, the model fit results should not be over interpreted and should therefore be interpreted with caution. Future research could further validate the measurement and structural model in independent samples, compare alternative model specifications, and examine whether a more parsimonious measurement structure can provide a better representation of the data.

Fourth, this study only examines the moderating role of team climate, and does not include other individual and situational variables that may be related to teaching innovation. Future research can further combine factors such as teaching self-efficacy, intrinsic motivation, school incentive system, digital teaching environment, and other factors to extend the research mode and more comprehensively reveal the multi-factor mechanism that affects the teaching innovation of university teachers.

Finally, the present study focused on examining influencing factors associated with teaching innovation behavior and did not address the design or implementation of specific support programs. Future research could build on the present findings to develop intervention programs centered on strengthening professional identity, enhancing teacher professional capital, and optimizing team climate, and could test their practical effects through quasi-experimental designs to provide actionable pathways for instructional reform in higher education.

## 7 Conclusion

Drawing on professional identity theory and conservation of resources theory as the theoretical basis, this study focused on Chinese university teachers and analyzed how professional identity, teacher professional capital, and team climate were statistically related to teaching innovation behavior. The findings support an integrated role-resource-context framework. Professional identity theory explains the role-value basis of innovative teaching, while conservation of resources theory explains how professional identity, as a personal resource, is connected with teaching innovation through professional resource accumulation and collaborative conditions. More specifically, university teachers with stronger professional identity tended to report more teaching innovation behavior, and part of this statistical link was accounted for by teacher professional capital. Team climate further served as a contextual condition, as the associations among professional identity, teacher professional capital, and teaching innovation behavior were stronger under a more favorable team climate. Overall, the study suggests that university teachers' teaching innovation behavior is linked not only to professional identity, but also to professional resource accumulation and team-based collaboration. Given the cross-sectional design, we should interpret these findings as correlational rather than causal. Future research may use longitudinal or intervention-based designs to further examine these relationships.

## Data Availability

The raw data supporting the conclusions of this article will be made available by the authors, without undue reservation.
